# The Book World of Medicine and Science

**Published:** 1904-12-24

**Authors:** 


### THE BOOK WORLD OF MEDICINE AND SCIENCE.

Malignant Disease of the Larynx. By P. de Santi, F.R.C S. (London: Baillière, Tindall and Cox. 1904. Pp. 107. Price 4s.).

This book contains a lucid and convincing exposition of what may be described as the English method of dealing with laryngeal cancer. Considerable emphasis is laid on the essential difference between intrinsic and extrinsic cancer of the larynx so far as the treatment and prognosis are concerned, and the operation of thyrotomy with excision of the disease, as practised by Butlin and Semon, is fully described, and its very great advantages over other measures are well demonstrated. At the same time the author takes a wide view, and does not fail to do justice to the good work of American and Continental surgeons. At the end of the book is a section dealing with sarcoma of the larynx. It may, perhaps, be regretted that no illustrations have been included, but beyond this there is little opportunity for adverse criticism. The book should be read by all who are interested in the subject of carcinoma of the larynx and its treatment.

The Surgical Treatment of Bright's Disease. By George M. Edebohls, M.D. (New York: Frank F. Lisiecki. 1904. Pp 327.)

The impression conveyed to the reader of this book is that Dr. Edebohls is too sanguine about the virtues of renal decapsulation. Certainly his cases fail to carry any conviction of the truth of his propositions. In the first place he includes under the heading of Bright's disease such conditions as albuminuria of pregnancy, and slight albuminuria accompanying and very possibly caused by undue mobility of one or both kidneys. It may be assumed that fixation of one or both kidneys in the latter case will prevent the congestion and consequent albuminuria, and if this assumption be granted, a fair number of Dr. Edebohl's successes will be accounted for. Again, the tendency toward complete recovery from the condition known as albuminuria of pregnancy is well known, and we do not think Dr. Edebohls should include these cases. Of the remainder, it is quite impossible in many instances to form any concept of the exact nature of the condition of the kidneys previous to operation, as the clinical and pathological descriptions may be justly described as slipshod. Of the 72 patients operated on, seven died within two weeks of the operation, 22 died at later periods, three disappeared from observation, and 40 are known to be living at periods varying from seven months to 12 years after the operation. In addition to the decapsulation of the kidneys, 63 other operations were performed on these 72 patients, including 13 operations for appendicitis. Seeing that chronic interstitial nephritis is a condition which may last many years we do not think that Dr. Edebohl's statistics warrant the inference that the condition is ameliorated by decapsulation; and we do not believe that the operation will be performed in this country for chronic nephritis until a more satisfactory exposition can be made in favour of Dr. Edebohl's procedure.

Biochemistry of Muscle and Nerve. By W. D. Halliburton, M.D., F.R.S., Professor of Physiology, King's College, London. (London: John Murray. Pp. 160. 33 illustrations. 1904. Price 7s. 6d. net.)

Professor Halliburton is well known as a pioneer in physiological chemistry, and the amount and quality of the research work which has issued from his laboratory is universally appreciated. The ten lectures of which the volume before us is comprised represent the substance of a course on the chemistry of muscle and nerve delivered in London in 1903 and repeated as the first course of Herter lectures in New York at the beginning of the present year. The style of the lecture theatre is retained, and the results of experiments conducted at the time are exhibited by reproductions of the actual tracings. Intricate as such biochemical problems, are their bearing on practical medicine is often a very direct one, as, for instance, the relation of the coagulation temperature of the globulin of the nerve-cells to the cause of fatal hyper-pyrexia; or the presence of choline in the blood, and cerebro-spinal fluid in general paralysis of the insane. The last chapter on the degeneration and regeneration of nerves after section is full of interest both for the physician and surgeon and is illustrated by some excellent photographs.

The Oxford and Cambridge Year-book, 1904. (London: Swan Sonnenschein and Co., Ltd. Two volumes. Price 3s. 6d. net per volume.)

The object of this year-book is to give particulars of the degrees, together with any other distinctions, and present occupation and address of all now living who have graduated either at Oxford or Cambridge. Despite the many difficulties of getting together such information the aim of the editor has been very satisfactorily effected, when it is considered that ninety per cent. of the entries in the book contain the required information and of the remaining ten per cent. some details at least are given although they are not complete. The book forms a very ready means of obtaining evidence of a university training and connection, and to all who require such information the work should prove exceedingly useful.

First Report of the Wellcome Research Laboratories at Khartoum. By Dr. A. Balfour. (1904. Department of Education, Khartoum.)

The research laboratories of the Gordon College were equipped by Mr. H. S. Wellcome, and are intended to serve several purposes, including the study of tropical diseases. The report before us deals with the work done in the latter province. As the laboratories were only opened in the early part of last year, it does very great credit to the management to have been able to produce a report at all, and especially does this remark apply to the able director, Dr. Balfour. The report contains articles on recent mosquito work in Egypt, biting and noxious insects other than mosquitos, and eosinophilia in bilharzia disease and dracontiasis. Included in the book are some very excellent coloured illustrations.

